# Concurrent Presentation of Idiopathic Intracranial Hypertension and Bilateral Optic Perineuritis in a Young Boy: A Rare Clinical Entity

**DOI:** 10.7759/cureus.102792

**Published:** 2026-02-01

**Authors:** Olga Vampertzi, Eleni Vouxinou, Asimina Mataftsi, Fotini Goutsaridou, Maria Fotoulaki, Dimitrios Zafeiriou

**Affiliations:** 1 4th Department of Pediatrics, School of Medicine, Faculty of Health Sciences, Papageorgiou General Hospital, Aristotle University of Thessaloniki, Thessaloniki, GRC; 2 2nd Department of Ophthalmology, School of Medicine, Faculty of Health Sciences, Papageorgiou General Hospital, Aristotle University of Thessaloniki, Thessaloniki, GRC; 3 Department of Radiology, School of Medicine, Faculty of Health Sciences, Papageorgiou General Hospital, Aristotle University of Thessaloniki, Thessaloniki, GRC; 4 1st Department of Pediatrics, School of Medicine, Faculty of Health Sciences, Hippokratio General Hospital, Aristotle University of Thessaloniki, Thessaloniki, GRC

**Keywords:** childhood, diplopia, optic perineuritis, papilledema, pseudotumor cerebri

## Abstract

Idiopathic intracranial hypertension (IIH) is a rare neurological syndrome characterized by increased intracranial pressure (>28 cm H₂O in children), in the absence of intracranial space-occupying lesions, and with normal cerebrospinal fluid (CSF) composition. Optic perineuritis (OPN) is an orbital inflammatory disease confined to the optic nerve sheath. We report a case of concurrent IIH and bilateral OPN in a previously healthy child.

A 13-year-old male, with normal body mass index, presented with diplopia, visual blurriness without ocular pain, and occipital headache. Neurological examination was notable only for right sixth nerve palsy. Ophthalmological assessment revealed bilateral papilledema. Brain and spinal cord magnetic resonance imaging (MRI) and computed tomography (CT) scans demonstrated bilateral optic nerve perineuritis. Lumbar puncture showed an opening pressure of 98 cm H₂O, with normal CSF composition. The patient was treated with acetazolamide and corticosteroids, resulting in marked clinical improvement and complete restoration of vision at two-month follow-up.

The concurrent occurrence of IIH and bilateral OPN is extremely rare, particularly in pediatric patients. To our knowledge, this is the first reported case in a child. Recognition of this dual pathology is essential for timely diagnosis and effective treatment.

## Introduction

Idiopathic intracranial hypertension (IIH), also known as pseudotumor cerebri (PTC), is an uncommon disorder in children and remains a diagnosis of exclusion. It is a benign neurological condition characterized by elevated intracranial pressure without an identifiable organic cause to explain clinical manifestations. The incidence of IIH in the general population ranges from 0.2 to 2 per 100,000, with a higher prevalence among obese women of childbearing age [1]. In the pediatric population, prevalence is estimated at 1:100,000-150,000 children, with approximately 60% of affected patients being older than 10 years. In children under six years of age, secondary causes of intracranial hypertension are more commonly identified [2,3]. The diagnostic criteria for IIH were first established by Dandy in 1937 [4], and subsequently revised by Friedman et al. in 2002 and 2013 [5]. Typical clinical features include headache, nausea, vomiting, and visual disturbances, often accompanied by papilledema, in an otherwise healthy child [6,7]. Despite extensive research, the pathophysiology underlying increased intracranial pressure in IIH remains incompletely understood.

Optic perineuritis (OPN) is a rare orbital inflammatory disorder affecting the optic nerve sheath. Initially described by Edmunds and Lawford in 1883 in patients with diabetes mellitus [8], it most commonly presents as acute, unilateral optic nerve dysfunction associated with ocular pain. Atypical cases, with bilateral, painless involvement, have also been reported [9,10]. OPN can be classified as primary (idiopathic) or secondary to systemic conditions. Secondary causes include demyelinating disorders, such as multiple sclerosis (MS), neuromyelitis optica spectrum disorder (NMOSD), and myelin oligodendrocyte glycoprotein (MOG)-IgG optic neuritis (ON). Autoimmune diseases, including systemic lupus erythematosus, sarcoidosis, and vasculitides, such as IgG4-related disease, granulomatosis with polyangiitis (GPA), giant-cell arteritis (GCA), and Behçet’s disease, may also contribute. Infectious etiologies, such as syphilis and tuberculosis, have additionally been implicated [11-14].

The coexistence of IIH and OPN is rare and poses a significant diagnostic challenge, as both may present with overlapping clinical and diagnostic features, including visual disturbances and optic disc edema. This challenge is particularly pronounced in the pediatric population, where the available literature data are limited, and accurate differentiation requires heightened clinical awareness.

In this report, we present a rare case of a 13-year-old boy with concurrent IIH and bilateral OPN, highlighting diagnostic difficulties and the importance of timely evaluation to guide appropriate, targeted therapy.

## Case presentation

A previously healthy, fully immunized 13-year-old male presented to the Emergency Department with diplopia, visual blurriness, headache, and vomiting. Symptoms began one week prior to admission, initially manifesting as blurred vision. Two days before presentation, he developed an occipital headache and neck pain, accompanied by vomiting and progressively worsening right esotropia, resulting in diplopia without ocular pain. Past neurological history was unremarkable, with no prior trauma, recent infections, or fever. Family history was non-contributory for neurological or autoimmune disorders, except for unilateral blepharoptosis in the father, who had no notable neurological or autoimmune abnormalities on examination.

On admission, vital signs were within normal limits, the Glasgow Coma Scale score was 15/15, and the body mass index was 21 kg/m². Physical examination revealed no additional abnormalities. Neurological assessment was notable only for right sixth cranial nerve palsy, manifested as esotropia and limitation of abduction of the right eye.

**Figure 1:**
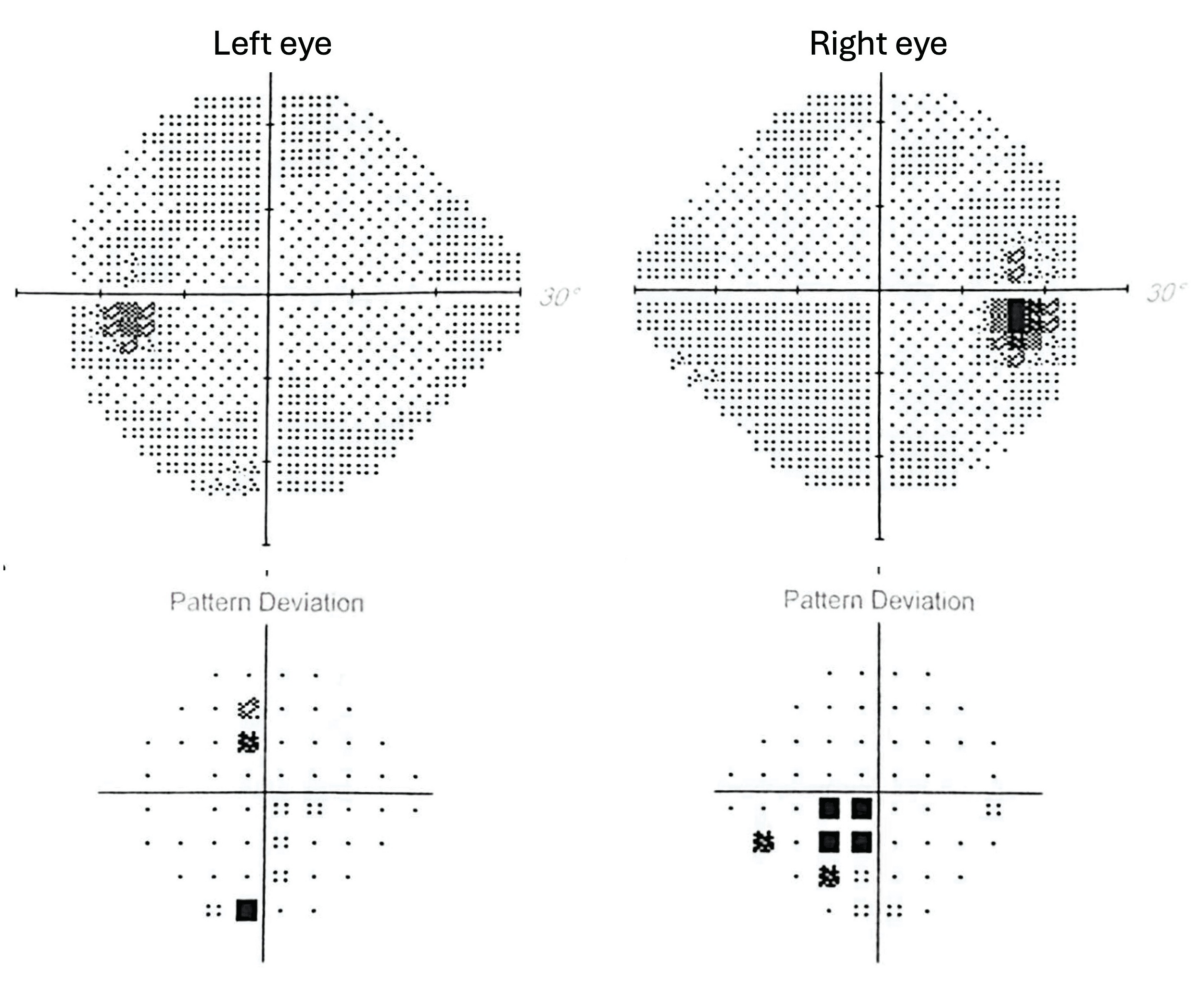
Automated visual field testing showing a scotoma in the lower nasal quadrant of the right eye.

**Figure 2:**
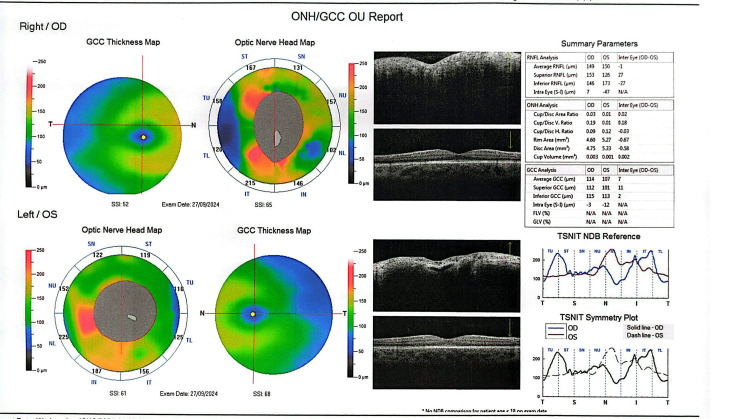
Optical coherence tomography (OCT) on admission demonstrating increased peripapillary retinal nerve fiber layer thickness, extensive bilateral optic disc swelling with peripapillary hemorrhages and paramacular atrophic changes.

Ophthalmological evaluation showed decreased visual acuity in the right eye (6/10). Automated central 30° visual field testing demonstrated a scotoma in the lower nasal quadrant of the right eye (Figure 1), and fundoscopic examination revealed bilateral papilledema with peripapillary hemorrhages (Figure 2).

The patient underwent an urgent brain computed tomography (CT) scan to exclude extrinsic compressive lesions or intracerebral hemorrhage. Imaging revealed bilateral enhancement of the optic nerves, suggestive of OPN, with otherwise normal brain parenchyma.

**Figure 3:**
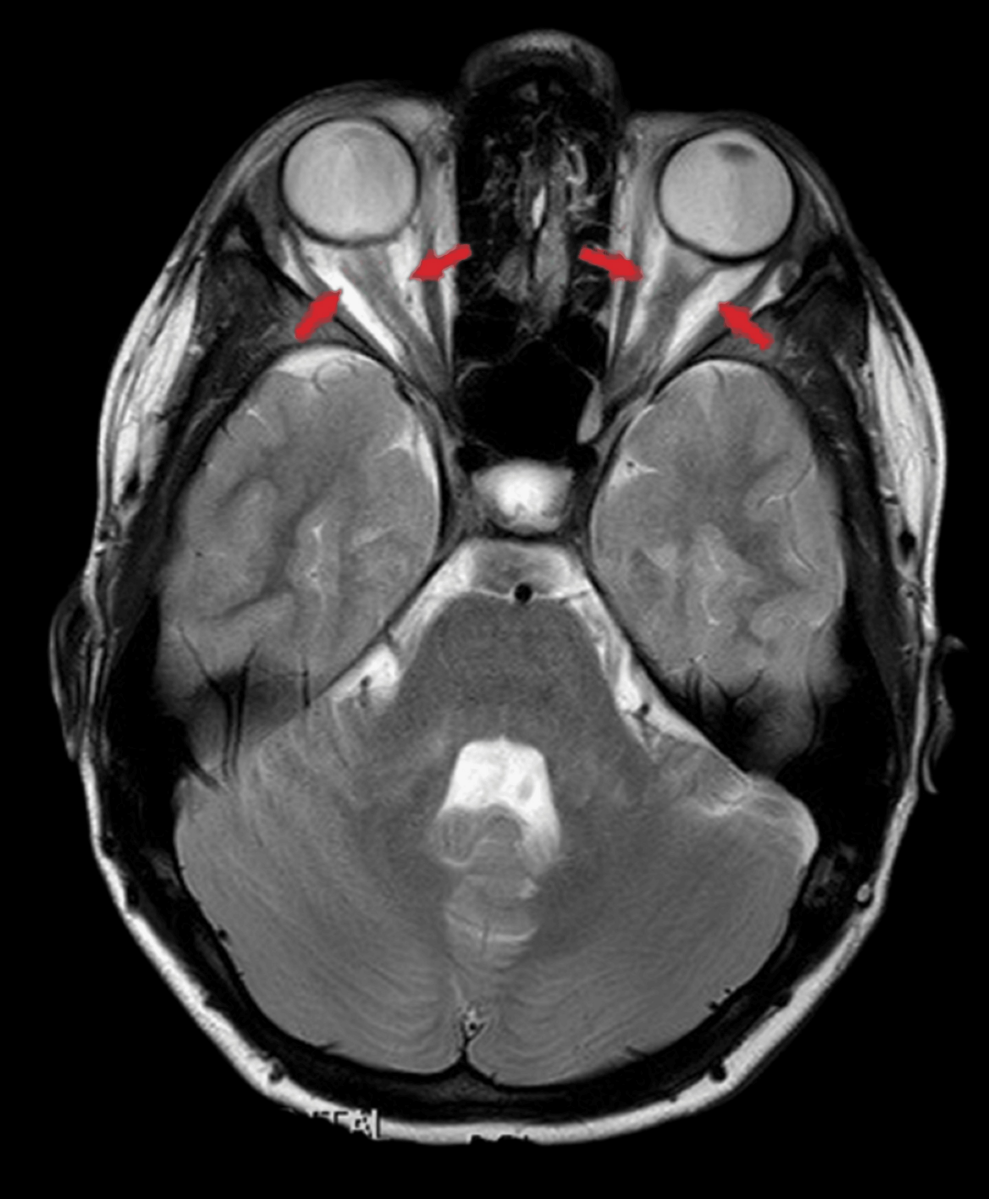
Axial contrast-enhanced magnetic resonance imaging (MRI) of the orbits showing bilateral circumferential enhancement of the optic nerve sheaths (Tram-Track sign, indicated by red arrows), consistent with optic perineuritis.

**Figure 4:**
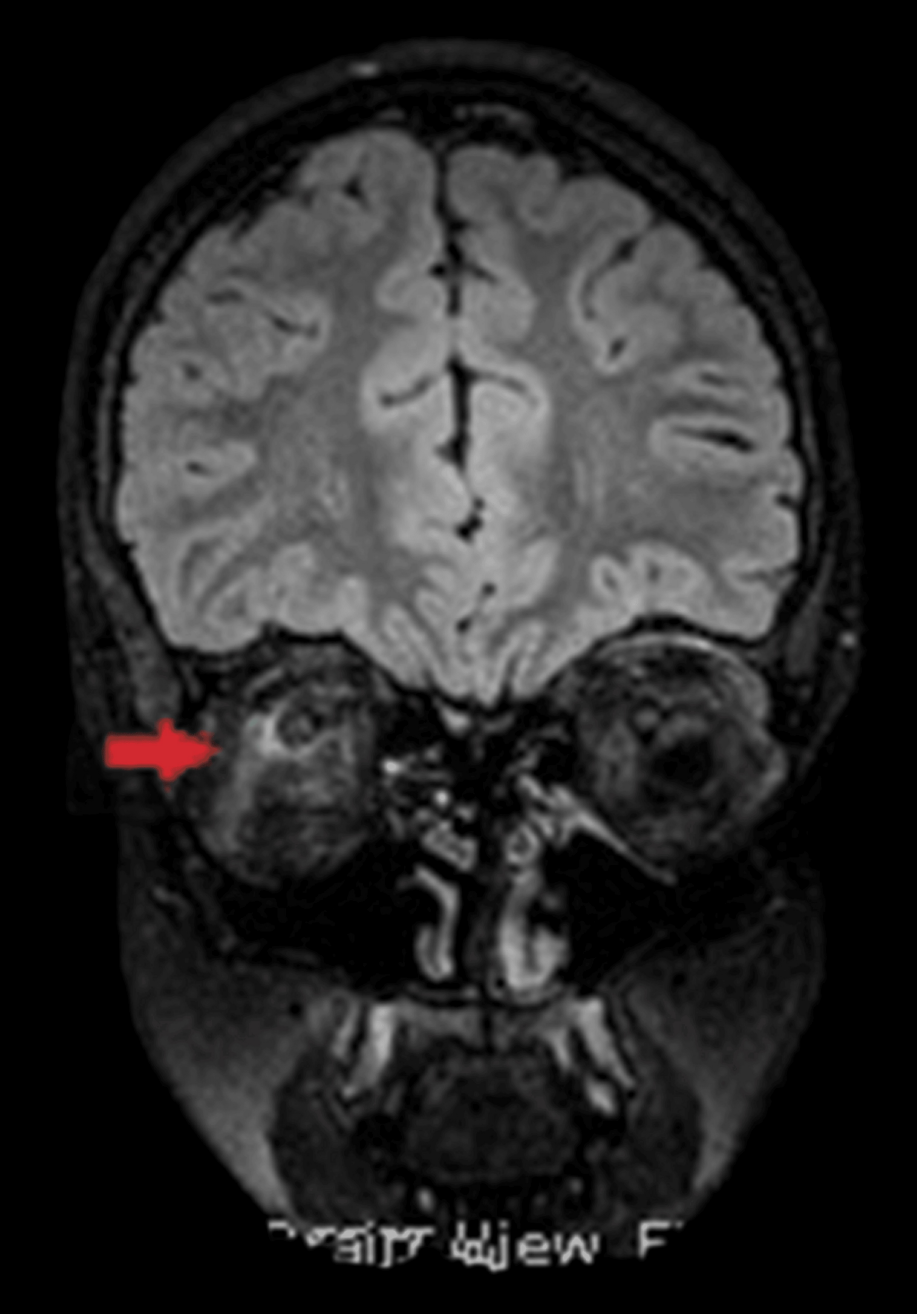
Coronal magnetic resonance imaging (MRI) of the orbits showing circumferential enhancement of the optic nerve sheath (Doughnut sign, indicated by red arrow).

Subsequent evaluation included intracranial magnetic resonance angiography (MRA) and venography (MRV), as well as spinal cord magnetic resonance imaging (MRI). MRA and MRV excluded major venous sinus thrombosis but demonstrated abnormal circumferential enhancement surrounding the intraorbital optic nerves, following intravenous contrast administration, consistent with optic nerve sheath inflammation. Additionally, MRI revealed a partially empty sella turcica without structural abnormalities or T2 signal changes, and hypoplasia of the left sigmoid and transverse venous sinuses (Figures 3-4). Spinal cord MRI was unremarkable.

Extensive laboratory testing was performed to exclude secondary causes of OPN and intracranial hypertension. Systemic lupus erythematosus (negative specific antibodies), sarcoidosis (normal angiotensin-converting enzyme levels), myasthenia gravis (negative acetylcholine receptor antibodies), and autoimmune vasculitis (negative antinuclear antibody (ANA), anti-double-stranded DNA (anti-dsDNA), and perinuclear antineutrophil cytoplasmic (p-ANCA) antibodies) were all excluded. Viral and bacterial infections were ruled out via serum antibodies and a FilmArray respiratory panel screening for 20 pathogens. Chest X-ray and tuberculin skin test were unremarkable. Electroencephalogram (EEG) showed no abnormal findings.

A lumbar puncture revealed markedly elevated opening pressure (98 cm H₂O) with normal cerebrospinal fluid (CSF) composition. CSF biochemical analysis and polymerase chain reaction (PCR) testing for 14 pathogens - including HSV-1 and HSV-2, varicella-zoster virus (VZV), human herpesvirus 6 (HHV-6), *Neisseria meningitidis*, and *Streptococcus pneumoniae* - were negative. CSF oligoclonal bands (OCBs) were negative, and serum antibodies against anti-MOG and aquaporin-4 (AQP4-IgG) were also negative, excluding MOG-IgG ON and NMOSDs. Based on these findings, a diagnosis of IIH and idiopathic bilateral OPN was established.

The primary treatment goal was to prevent vision loss. The patient received acetazolamide, gradually titrated to 20 mg/kg/day, combined with oral potassium supplementation. High-dose intravenous methylprednisolone (1,000 mg/day for three days) was administered, followed by oral prednisolone (1 mg/kg/day) with gradual tapering over 30 days. Symptomatic improvement was noted immediately after lumbar puncture, with resolution of headache and vomiting. Diplopia, visual blurriness, and esotropia improved progressively, with complete resolution within 20 days.

**Figure 5:**
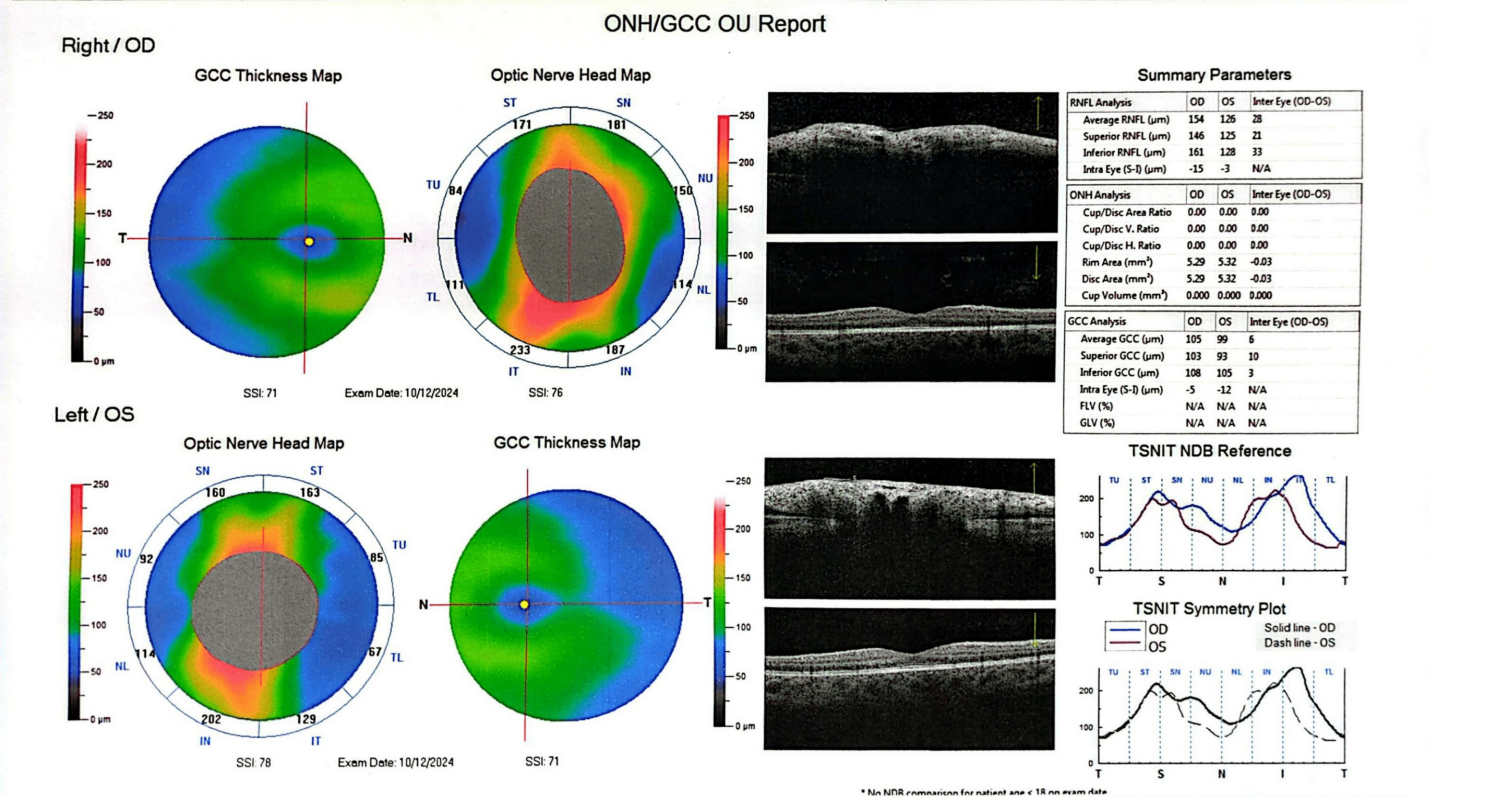
Optical coherence tomography (OCT) at two-month follow-up showing improvement of optic disc edema and reduced peripapillary subretinal fluid.

**Figure 6:**
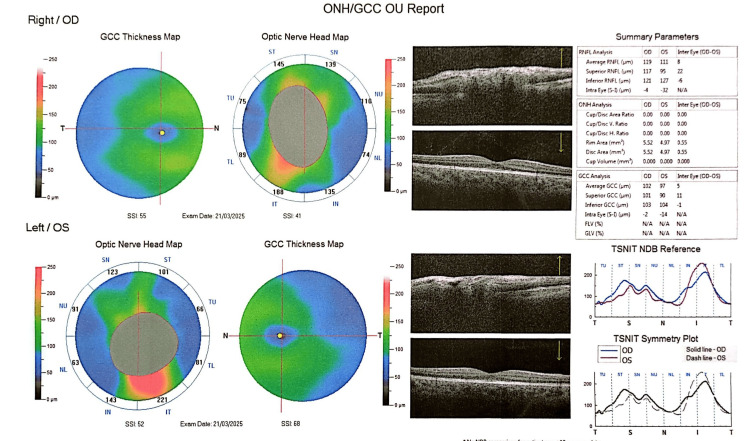
Optical coherence tomography (OCT) at six-month follow-up demonstrating complete resolution of papilledema in both eyes.

The patient remains under regular follow-up, demonstrating excellent clinical response. Due to acetazolamide-induced metabolic acidosis, he currently receives sodium bicarbonate supplementation. At two-month follow-up, optical coherence tomography (OCT) revealed improvement of optic disc edema and reduced peripapillary subretinal fluid (Figure 5). At six months, fundoscopy and OCT demonstrated complete remission of papilledema in both eyes (Figure 6).

## Discussion

The diagnosis of pediatric IIH relies on lumbar puncture demonstrating an opening pressure exceeding 28 cm H₂O, or greater than 25 cm H₂O in non-obese, non-sedated children, according to the revised diagnostic criteria by Friedman et al. [5]. In the present case, although the patient had a normal body mass index, his lumbar puncture revealed a markedly elevated opening pressure of 98 cm H₂O.

He fulfilled all modified Dandy criteria for a definitive diagnosis of PTC syndrome, which include normal neurological examination except for sixth cranial nerve palsy, bilateral papilledema, normal CSF biochemical composition, and normal brain parenchyma on MRI [5].

Pediatric IIH is a rare disorder characterized by elevated intracranial pressure without structural brain lesions or abnormal CSF composition. In the present case, intracranial MRA and MRV excluded structural abnormalities such as meningioma or hydrocephalus and demonstrated findings consistent with IIH, including a partially empty sella, distension of the perioptic subarachnoid space, and transverse venous sinus stenosis - recognized neuroimaging criteria for IIH even in patients without papilledema [1].

Headache is the most frequent presenting symptom in pediatric IIH, reported in 57%-87% of cases, particularly among older children and adolescents [15]. The characteristics of headache in pediatric IIH are heterogeneous, ranging from diffuse to focal pain, and are often exacerbated by maneuvers that increase intracranial pressure, such as coughing or sneezing [16]. In children, headache frequently radiates to the neck, reflecting increased sensitivity of the spinal nerve root dural sheaths in the setting of elevated intracranial pressure [16]. In the present case, the patient experienced a sudden-onset occipital headache and neck pain, consistent with these typical clinical features and accompanied by nausea and vomiting.

Visual disturbances are a common feature in pediatric IIH, with diplopia representing the second most frequent symptom after headache [6,17]. Papilledema occurs due to increased pressure within the optic nerve sheath, leading to venous stasis and accumulation of extracellular fluid at the optic disc [18]. The underlying pathophysiology of IIH remains incompletely understood, with proposed mechanisms including increased CSF production, elevated cerebral blood volume, and impaired CSF absorption, secondary to venous outflow obstruction. In this patient, hypoplasia of the left transverse and sigmoid sinuses may have contributed to impaired venous drainage and elevated intracranial pressure [19,20].

A diagnostic challenge in this case was the presence of bilateral optic nerve sheath enhancement on orbital MRI, consistent with OPN. Clinically, bilateral painless visual impairment and diplopia raised the differential diagnosis of ON, which typically presents as unilateral, acute, painful vision loss and is often associated with MS [21]. Recognition of these overlapping clinical features is crucial for timely diagnosis and appropriate management.

Although the diagnosis of IIH was established, it is important to consider demyelinating disorders in cases of bilateral optic nerve involvement. NMOSD and MOG-IgG-associated ON can present with significant papilledema and bilateral visual impairment [21]. In this patient, serum AQP4-IgG, anti-MOG antibodies, and CSF OCBs were all negative, and spinal MRI excluded longitudinally extensive lesions, typical of Devic’s disease [22,23]. Such evaluations are essential, as cases of fulminant IIH have been reported in which MOG-IgG-associated ON was initially overlooked [22]. Comprehensive diagnostic workup ensures accurate differentiation between IIH and other optic neuropathies, guiding appropriate management and preventing vision-threatening complications.

On the other hand, OPN is a rare inflammatory disorder of the optic nerve sheath, first described by Dutton and Anderson in 1985 as a chronic granulomatous inflammation that can lead to circumferential compression of the optic nerve and secondary ischemic injury [24]. Although often idiopathic, OPN has been associated with systemic inflammatory and autoimmune conditions, including systemic lupus erythematosus, ANCA-associated vasculitis, sarcoidosis, and MOG-IgG-associated demyelinating disease, as well as infectious etiologies such as tuberculosis, syphilis, herpes viruses, and toxoplasmosis [14,25,26]. Clinically, OPN can closely mimic ON, typically presenting with subacute visual impairment and retro-orbital pain, often exacerbated by eye movements [27]. However, painless, subacute visual deterioration, as observed in this patient, has also been reported in OPN. While OPN is usually unilateral, bilateral involvement has been described in the literature [28]. Fundoscopic findings, such as optic disc edema and visual field defects, including paracentral scotomata, may be present in both OPN and IIH, which can complicate clinical differentiation [14]. Recognizing these overlapping features is critical for accurate diagnosis and timely management.

OCT is a valuable tool for monitoring optic disc edema in patients with OPN or IIH, as increased peripapillary retinal nerve fiber layer thickness reflects papilledema [27]. However, OCT alone does not allow differentiation between underlying etiologies. In the present case, OCT was particularly useful during follow-up, demonstrating significant improvement of optic disc swelling after two months of treatment. Definitive diagnosis of OPN relies on characteristic orbital MRI findings, with enhancement of the optic nerve sheath and relative sparing of the optic nerve itself, indicative of perioptic subarachnoid space enlargement. Classic imaging features, including the “tram-track” sign on axial images and the “doughnut” sign on coronal views, are considered pathognomonic and were observed in this patient [11]. Optic nerve biopsy is rarely required and is generally reserved for refractory cases with poor response to corticosteroid therapy [14]. Recognition of these imaging features is critical for accurate diagnosis, timely initiation of therapy, and prevention of irreversible visual impairment.

Systemic corticosteroids remain the mainstay of treatment for OPN in patients with preserved visual acuity, often leading to rapid clinical improvement [5]. Although spontaneous resolution has been reported, early intervention is generally recommended to prevent irreversible optic nerve damage [29].

To date, only three cases of concurrent bilateral OPN and IIH have been documented. The first involved a 38-year-old obese woman with recent COVID-19 infection, whose visual acuity improved only after treatment with intravenous immunoglobulin and acetazolamide [30]. The second case described an 18-year-old obese girl with pre-existing IIH, who developed painless bilateral visual blurring and achieved partial visual recovery following corticosteroid and acetazolamide therapy at one-year follow-up [31]. The third case, reported by Bellucci et al., involved a 62-year-old overweight woman with severe headache and visual blurring; bilateral OPN was diagnosed by orbital MRI, and corticosteroid therapy resulted in gradual improvement of vision and headache resolution [32].

In contrast, the present patient represents a rare pediatric case with simultaneous IIH and bilateral OPN. Notably, combined therapy led to marked visual recovery within two months, emphasizing the importance of early recognition, comprehensive diagnostic evaluation, and prompt management in atypical pediatric presentations.

## Conclusions

Idiopathic bilateral OPN is an exceptionally rare condition, typically reported in older adults and predominantly affecting women. In this report, to our knowledge, we describe the first adolescent male with concurrent bilateral OPN and IIH. The patient had a normal body mass index, in contrast to the obesity often seen in pediatric IIH, highlighting the need for clinical vigilance even in patients without typical comorbidities. Early recognition and prompt combined therapy with acetazolamide and systemic corticosteroids were associated with rapid visual improvement in this patient. While exceedingly uncommon, this case illustrates that OPN and IIH can coexist in pediatric patients and should be considered in the differential diagnosis of children presenting with visual disturbances and signs of raised intracranial pressure.

To our knowledge, this case report is particularly valuable, as it represents the first description of idiopathic bilateral OPN in a pediatric patient associated with IIH; given the diagnostic challenges and the risk of irreversible complications, including permanent vision loss, heightened awareness and prompt management are essential within the pediatric community, even for such rare presentations.

## References

[1] Rohit W, Rajesh A, Mridula R, Jabeen SA (2021). Idiopathic intracranial hypertension - challenges and pearls. Neurol India.

[2] Malem A, Sheth T, Muthusamy B (2021). Paediatric idiopathic intracranial hypertension (IIH): a review. Life (Basel).

[3] Balbi GG, Matas SL, Len CA, Fraga MM, Sousa IO, Terreri MT (2018). Pseudotumor cerebri in childhood and adolescence: data from a specialized service. Arq Neuropsiquiatr.

[4] Dandy WE (1937). Intracranial pressure without brain tumor: diagnosis and treatment. Ann Surg.

[5] Friedman DI, Liu GT, Digre KB (2013). Revised diagnostic criteria for the pseudotumor cerebri syndrome in adults and children. Neurology.

[6] Hacifazlioglu Eldes N, Yilmaz Y (2012). Pseudotumour cerebri in children: etiological, clinical features and treatment modalities. Eur J Paediatr Neurol.

[7] Incecik F, Hergüner MO, Altunbaşak S (2011). Evaluation of sixteen children with pseudotumor cerebri. Turk J Pediatr.

[8] Edmunds W, Lawford J (1883). Examination of optic nerve from cases of amblyopia in diabetes. Trans Ophthalmol Soc UK.

[9] Zaidi S (2013). Optic perineuritis: uncommon presentation of more common diseases. J Neurol Neurosurg Psychiatry.

[10] Tevaraj JM, Tai Li Min E, Mohd-Noor RA, Thavaratnam LK, Salmah WM, Wan Hitam WH (2016). Atypical presentation of idiopathic bilateral optic perineuritis in a young patient. Case Rep Ophthalmol Med.

[11] Purvin V, Kawasaki A, Jacobson DM (2001). Optic perineuritis: clinical and radiographic features. Arch Ophthalmol.

[12] Bergman O, Andersson T, Zetterberg M (2017). Optic perineuritis: a retrospective case series. Int Med Case Rep J.

[13] McClelland C, Zaveri M, Walsh R, Fleisher J, Galetta S (2012). Optic perineuritis as the presenting feature of Crohn disease. J Neuroophthalmol.

[14] Gupta S, Sethi P, Duvesh R, Sethi HS, Naik M, Rai HK (2021). Optic perineuritis. BMJ Open Ophthalmol.

[15] Barmherzig R, Szperka CL (2019). Pseudotumor cerebri syndrome in children. Curr Pain Headache Rep.

[16] Hamedani AG, Witonsky KF, Cosico M (2018). Headache characteristics in children with pseudotumor cerebri syndrome, elevated opening pressure without papilledema, and normal opening pressure: a retrospective cohort study. Headache.

[17] Matthews YY, Dean F, Lim MJ (2017). Pseudotumor cerebri syndrome in childhood: incidence, clinical profile and risk factors in a national prospective population-based cohort study. Arch Dis Child.

[18] Hayreh SS (2016). Pathogenesis of optic disc edema in raised intracranial pressure. Prog Retin Eye Res.

[19] Cleves-Bayon C (2018). Idiopathic intracranial hypertension in children and adolescents: an update. Headache.

[20] Albakr A, Hamad MH, Alwadei AH (2016). Idiopathic intracranial hypertension in children: diagnostic and management approach. Sudan J Paediatr.

[21] Bennett JL (2019). Optic neuritis. Continuum (Minneap Minn).

[22] Wendel EM, Tibussek D, Barisic N (2025). Children with MOG-IgG positive bilateral optic neuritis misdiagnosed as fulminant idiopathic intracranial hypertension. Mult Scler Relat Disord.

[23] Kim HJ, Paul F, Lana-Peixoto MA (2015). MRI characteristics of neuromyelitis optica spectrum disorder: an international update. Neurology.

[24] Dutton JJ, Anderson RL (1985). Idiopathic inflammatory perioptic neuritis simulating optic nerve sheath meningioma. Am J Ophthalmol.

[25] Saitakis G, Chwalisz BK (2022). Optic perineuritis. Curr Opin Ophthalmol.

[26] Li H, Zhou H, Sun J, Wang H, Wang Y, Wang Z, Li J (2020). Optic perineuritis and its association with autoimmune diseases. Front Neurol.

[27] Phillips PH, Sheldon CA (2017). Pediatric pseudotumor cerebri syndrome. J Neuroophthalmol.

[28] El-Bouz M, Msaaf H, Aoued L, Gueddari W (2024). Rare cause of pseudotumor cerebri in children. Pediatr Oncall.

[29] Tung C, Hashemi N, Lee AG (2013). Spontaneous resolution of optic perineuritis. J Neuroophthalmol.

[30] Sardar S, Safan A, Okar L, Sadik N, Adeli G (2021). The diagnostic dilemma of bilateral optic neuritis and idiopathic intracranial hypertension coexistence in a patient with recent COVID-19 infection. Clin Case Rep.

[31] Shahrudin NH, Muhammed J, Wan Hitam WH, Sapiai NA, Abdul Halim S (2024). A case report of bilateral optic perineuritis with idiopathic intracranial hypertension: challenges in diagnosis and management. Cureus.

[32] Bellucci G, De Riggi M, Di Bonaventura C, Suppa A, Leodori G, Fiorelli M, Fabbrini G (2024). Blurred lines: bilateral optic perineuritis mimicking idiopathic intracranial hypertension. Neurol Sci.

